# Continuous monitoring of patients in and after the acute admission ward to improve clinical pathways: study protocol for a randomized controlled trial (Optimal-AAW)

**DOI:** 10.1186/s13063-023-07416-8

**Published:** 2023-06-15

**Authors:** Sjoerd H. Garssen, Niels Kant, Carlijn A. Vernooij, Gert-Jan Mauritz, Mark V. Koning, Frank H. Bosch, Carine J. M. Doggen

**Affiliations:** 1 Clinical Research Center, Rijnstate Hospital, Arnhem, The Netherlands; 2 Department of Patient Care and Monitoring, Philips Research, Eindhoven, The Netherlands; 3 Department of Health Technology and Services Research, Technical Medical Center, University of Twente, Enschede, The Netherlands; 4 Department of Anesthesiology, Rijnstate Hospital, Arnhem, The Netherlands; 5 Department of Emergency Medicine, Rijnstate Hospital, Arnhem, The Netherlands; 6 Department of Internal Medicine, Rijnstate Hospital, Arnhem, The Netherlands; 7 Department of Internal Medicine, Radboudumc, Nijmegen, The Netherlands

**Keywords:** Wearable electronic device, Acute admission ward, Length of stay, Patient discharge, Clinical trial protocol, Monitoring, physiologic, Clinical deterioration, Clinical decision rules

## Abstract

**Background:**

Because of high demand on hospital beds, hospitals seek to reduce patients’ length of stay (LOS) while preserving the quality of care. In addition to usual intermittent vital sign monitoring, continuous monitoring might help to assess the patient’s risk of deterioration, in order to improve the discharge process and reduce LOS. The primary aim of this monocenter randomized controlled trial is to assess the effect of continuous monitoring in an acute admission ward (AAW) on the percentage of patients who are discharged safely.

**Methods:**

A total of 800 patients admitted to the AAW, for whom it is equivocal whether they can be discharged directly after their AAW stay, will be randomized to either receive usual care without (control group) or with additional continuous monitoring of heart rate, respiratory rate, posture, and activity, using a wearable sensor (sensor group). Continuous monitoring data are provided to healthcare professionals and used in the discharge decision. The wearable sensor keeps collecting data for 14 days. After 14 days, all patients fill in a questionnaire to assess healthcare use after discharge and, if applicable, their experience with the wearable sensor. The primary outcome is the difference in the percentage of patients who are safely discharged home directly from the AAW between the control and sensor group. Secondary outcomes include hospital LOS, AAW LOS, intensive care unit (ICU) admissions, Rapid Response Team calls, and unplanned readmissions within 30 days. Furthermore, facilitators and barriers for implementing continuous monitoring in the AAW and at home will be investigated.

**Discussion:**

Clinical effects of continuous monitoring have already been investigated in specific patient populations for multiple purposes, e.g., in reducing the number of ICU admissions. However, to our knowledge, this is the first Randomized Controlled Trial to investigate effects of continuous monitoring in a broad patient population in the AAW.

**Trial registration:**

https://clinicaltrials.gov/ct2/show/NCT05181111. Registered on 6 January 2022. Start of recruitment: 7 December 2021.

**Supplementary Information:**

The online version contains supplementary material available at 10.1186/s13063-023-07416-8.

## Introduction

As the demand on hospital beds increases over the years [1] and the shortage of nurses continues [2], hospitals aim to increase efficacy. Therefore, patients are discharged as early as possible when it is medically safe [3]. In the Netherlands, patients presented in an Emergency Department (ED) are commonly admitted, either for specific treatment or for further observation, to an acute admission ward (AAW). This non-intensive ward, commonly referred to as an Acute Medical Unit, has been widely implemented in multiple countries [4]. It is a ward where patients from different specialties in need of acute care are admitted to for typically 24 to 72 h prior to discharge or transfer to an in-hospital ward. During this intentionally short stay in the AAW, a patient’s risk of deterioration is assessed by intermittent vital sign monitoring and the calculation of a corresponding Early Warning Score (EWS). The frequency of this assessment is determined by the patient’s condition and typically performed every 4 to 8 h. Although for some patients the risk of deterioration is clearly low or high, for other patients it is uncertain whether deterioration will occur. Subsequently, patients with this uncertainty are often admitted to an in-hospital ward. However, the need for such an admission to an in-hospital ward is equivocal. For patients that do require in-hospital care, early detection of stability may decrease the uncertainty regarding hospitalization and provide care planners with the opportunity to allocate a bed in a suitable ward in a timely manner. Consequently, these patients are expected to be admitted to the appropriate ward sooner, potentially reducing their length of stay (LOS) in the AAW. For patients who can be discharged, the time spent in the AAW may also be reduced due to earlier confirmation of stability. The reductions of LOS and unnecessary admissions to in-hospital wards might increase the efficacy of the use of hospital beds.

The EWS consists of several parameters that may predict deterioration, of which heart rate (HR) and respiratory rate (RR) are especially predictive [5–7]. Continuous monitoring of these vital signs, which can be facilitated by wearable sensors, may therefore help to timely assess safe discharge [8]. Such sensors measure vital signs wirelessly and continuously, without reducing patient’s mobility and with potentially decreasing nurses’ workload [9–12]. Although several wearable sensors have been validated and are increasingly used in surgery wards, the use of these devices to optimize the discharge process in the AAW has not been investigated before [13, 14]. Hence, proof of clinical benefit and cost-effectiveness is still lacking [15, 16].

Therefore, the primary aim of this randomized controlled trial is to assess the effect of continuous monitoring of HR and RR on the percentage of patients who can be safely discharged directly from the AAW. Safe discharge is defined by the absence of unplanned readmissions, ED revisits, or mortality occurrences within 30 days.

## Methods

### Study setting

This trial (NCT05181111—ClinicalTrials.gov) is a monocenter randomized controlled trial with two groups and is conducted in Rijnstate Hospital, Arnhem, the Netherlands. Rijnstate is a 766-bed community teaching hospital covering an area of 450,000 inhabitants. This hospital, like most Dutch hospitals, has an AAW where patients are temporarily monitored using intermittent vital sign monitoring to assess the risk of deterioration and enhance patient flow from the ED [17]. In accordance with the local hospital protocol, all patients in need of hospitalization from the ED could be admitted to the AAW, except when patients meet certain exclusion criteria (see Table 1). Consequently, the AAW accommodates a diverse patient population, including both surgical and non-surgical patients. In 2019, there were 9077 admissions to the AAW in Rijnstate. In this trial, participating patients will be randomized to a control group receiving only usual care or to a sensor group receiving usual care with an additional wearable sensor for continuous monitoring, which is described in more detail in the intervention section. This trial protocol uses the Standard Protocol Items: Recommendations for Interventional Trials (SPIRIT) reporting guidelines (see Additional file 1) [18].

**Table 1:** Exclusion criteria for admission to the acute admission ward of Rijnstate Hospital

Exclusion criteria for admission to the acute admission ward.	
**General**	- In need of admittance to the cardiac care unit, medium care, or intensive care - Requiring specific psychiatric care - 18 years old - Multiple fractures - In need of isolation (tuberculosis, MRSA, varicella zoster) - Requiring post-examination recovery
**Internal Medicine**	- Lymphatic leukemia - HIV that requires treatment - In need of dialysis - Renal transplant - Autologous stem cell transplantation within the last 6 months
**Pulmonology**	- In need of non-invasive ventilation - In need of >5 l/min of supplemental oxygen - Unstable after 2 h of oxygen supply via non-rebreathing mask
**Neurology**	- Only patients with minor head trauma are accepted
**Gastroenterology**	- Pancreatitis - Gastrointestinal bleeding - Choledocholithiasis and cholangitis - Inflammatory bowel disease - Gastroenterological malignancy - Liver cirrhosis
**Urology**	- Severe hematuria - Terminal disease
**Otorhinolaryngology**	- Cuffed tracheal cannula - In need of advanced airway care - Severe facial fractures
**Geriatrics**	- Severe delirium - Terminal disease

### Study population

All ED patients who are scheduled to be admitted to the AAW are screened by emergency physicians to identify those eligible for participation in this trial. This trial aims to recruit patients whose discharge destination following the AAW is uncertain upon admission, in order to effectively evaluate the impact of continuous monitoring on the discharge process. The emergency physician identifies these patients by answering the following questions: Is it certain that the patient will be discharged within the next 24 h? Is it certain that, after admission to the AAW, the patient must be transferred to an in-hospital ward? When both questions are answered “no,” the patient is considered an “equivocal patient” and is eligible for participation. Other inclusion criteria are that patients should be 18 years or older and are able to speak and read Dutch. Exclusion criteria are known pregnancy, breastfeeding, not able or willing to wear a wearable sensor for 14 days, scheduled surgery within the next 30 days, an active implantable device, any skin condition where the wearable sensor needs to be placed, known sensitivity to medical adhesives, use of creams or lotions that influence the skin at the area where the wearable sensor is placed or intend to go the sauna or swimming in the next 14 days. Patients with a pacemaker or known pregnancy are excluded from participation due to the potential influence of the pacemaker or child on the sensor measurements, respectively. Breastfeeding may be hindered by the sensor, which was deemed undesirable. Patients scheduled for surgery within 30 days are excluded as it may directly impact the primary outcome measure due to prolonged or new hospitalizations after surgery. Following the instruction for use, the manufacturer advises against swimming or visiting the sauna, as this may lead to the detachment of the sensor.

### Outcomes

The primary outcome is the difference between the control and sensor group in the percentage of patients who are safely discharged from the hospital directly after AAW stay. A safe discharge is defined as a discharge without following unplanned hospital readmission, ED revisit, or mortality within 30 days. Secondary outcomes are LOS in the AAW, LOS in the in-hospital wards, percentage of RRT calls during AAW and hospital stay, percentage of ICU admissions during AAW and hospital stay, and percentage of unplanned readmissions to the hospital within 30 days after hospital discharge. Furthermore, facilitators and barriers for implementing continuous monitoring in usual care in the AAW will be assessed by questionnaires and interviews with healthcare professionals. Also, for the sensor group, the feasibility of remote home monitoring with the wearable sensor is assessed, including the duration for which the sensor was worn, factors leading to its early detachment, user complaints regarding wearing the sensor, and restrictions in daily activities.

### Statistics

For the descriptive analyses (e.g., characteristics of participants), continuous variables will be reported as mean with standard deviation for normally distributed data, and median with interquartile range for non-normally distributed data. For categorical variables, numbers and percentages will be reported.

For the primary outcome, the proportion of patients discharged home safely, directly after AAW stay, will be compared between the control and sensor group using a chi-squared test. Differences in LOS will be tested using Student’s *t*-test or Mann-Whitney test depending on the presence of a normal distribution. To assess whether the percentages of ICU admissions, RRT calls, and hospital admissions differ between the control and sensor groups, chi-squared or Fisher’s exact tests will be used, as appropriate. Intention-to-treat analyses will be used for the primary and secondary outcomes. Additionally, per-protocol analyses will be used to assess the effect of the sensor, adjusted for patients who receive a non-functional sensor due to technical reasons. All remaining patients will be included in these per-protocol analyses, regardless of their destination after AAW admission and the duration for which the wearable sensor was worn. An interim analysis for futility is planned once 50% of total amount of patients is included.

### Sample size

Based on a preliminary internal investigation and insights of healthcare professionals working in the AAW, the proportion of the control group that is safely discharged was estimated to be 40%. As an increase to 50% was deemed to be feasible and clinically relevant, this proportion was used for the sensor group in calculating the sample size. The aim is to reject the null hypothesis stating that the proportion of patients that are discharged home safely directly after AAW stay is equal in the control and sensor groups. With a power of 80%, an *α* of 0.05, and equal group sizes, a total of 768 participants are required [19]. To achieve this number of patients with an assumed drop-out rate of 5%, the sample size is set to 800 patients, i.e., 400 in the control group and 400 in the sensor group.

### Recruitment

When all criteria for participation are met, the patient receives verbal and written information about the trial from an emergency physician at the ED. The patient is able to consider participation and ask questions to any physician or nurse until 2 h after admission to the AAW. If a patient decides to participate in the trial, the patient signs an informed consent form. Subsequently, the patient will be allocated to either the control or sensor group. Participants cannot switch between the control and sensor groups. However, a patient is allowed to withdraw from the trial without providing a reason for withdrawal and still receives usual care. Data generated of these patients will be used for analyses until the date of withdrawal, unless a patient revokes informed consent completely.

### Randomization

This trial will employ a 1:1 randomization scheme with block sizes of four and eight, in random order, to allocate patients to either the control or sensor group. A computer-generated randomization scheme is consulted by a nurse after the patient has provided informed consent.

### Blinding

The allocation of patients is concealed from both the patient and the nurse. After allocation, both nurse and patient are aware of the patient’s allocation due to the attachment of a wearable sensor generating real-time data. Outcome assessors (SG, NK) are not blinded to facilitate study logistics. However, because the outcomes cannot be influenced by the outcome assessors, any potential bias attributable to the absence of blinding is considered negligible. Data analysts (SG, NK) are also not blinded in this trial.

### Wearable sensor

As a wearable sensor, the “Healthdot” (Philips Electronics Nederland B.V.) is used [20]. This is a wearable patch of five to three centimeters that weighs 13.6 g. The device is attached to the left lower rib of the patient on the mid-clavicular line. The accelerometer-based device uses seismocardiography and displacement along different axes to calculate HR, RR, posture, and activity levels every 5 min [21, 22]. It then transmits these data directly to a cloud via a low-power wide-area network (LoRaWAN), from where it can be transferred to the hospital IT infrastructure. The real-time data of measurements are displayed in tabular and graphical form on a dashboard called IntelliVue Guardian Software (version E.01.00) and are available for healthcare professionals on their computer desktop after logging in.

The conventional EWS used in the AAW uses RR, HR, oxygen saturation, oxygen supply, systolic blood pressure, consciousness, and temperature with predetermined thresholds to calculate a EWS between 0 and 20. The data from the wearable sensor are used to automatically calculate a sensor EWS solely based on HR and RR, which uses the same thresholds as the conventional EWS, resulting in a sensor EWS between 0 and 6. This score is converted to a risk band, which can be green (EWS ≤ 1), orange (EWS 2), or red (EWS >2), indicating the risk of deterioration. In case the risk band remains increased for three subsequent periods of 15 min, a notification is displayed on the dashboard. This notification can be visualized by nurses and physicians in the AAW when logged into the dashboard. The nurses and physicians of the AAW are free to act upon these notifications, for instance by performing an additional check-up. When a patient remains within the same risk band or improves within 45 min, no notification is displayed.

The wearable sensor is employed for the full extent of time specified by the manufacturer, which is 14 days. It is possible that a patient in the sensor group wears the wearable sensor for less than 14 days. Possible reasons are accidental detachment; removal due to unexpected side effects, such as skin rash; removal by a healthcare provider for other reasons; or removal due to patient withdrawal. In these cases, there is no replacement for the wearable sensor. Reattaching the wearable sensor is not possible because the adhesive layer is designed for single use only. If a wearable sensor is detached while the patient is admitted to the AAW, a new wearable sensor is attached if permitted by the patient. In line with the instructions for use, detachment is only necessary when a patient needs an MRI scan. To prevent unnecessary detachment of the wearable sensor after hospital discharge, patients receive an information card with instructions for healthcare providers in case the healthcare provider is in doubt as to whether a medical interference requires the removal of the sensor.

### Data collection and management

Data are collected from four different sources. Firstly, data on medical treatment, laboratory tests, patient characteristics, and primary and secondary outcomes, such as whether a patient was safely discharged, are retrieved from the Electronic Medical Record (EMR). Secondly, for the sensor group, wearable sensor data of the overall period up to 14 days are collected. Thirdly, supplementary data pertaining to the primary outcome, specifically healthcare usage after discharge (e.g., readmissions in other hospitals), will be obtained from patient questionnaires. The questionnaires also include information regarding the patient’s experience with the wearable sensor to assess feasibility. Since there is currently no validated questionnaire aligned with our study objectives, self-constructed questionnaires will be employed. Fourthly, data from questionnaires and interviews with healthcare professionals are collected at the end of the trial. Healthcare professionals will be selected using a stratified sampling method, ensuring a representative distribution of all specialties that operate in the AAW. These data will be used to investigate facilitators and barriers for implementing continuous monitoring in usual care in the AAW.

All collected data are protected according to data protection standards of the Netherlands and the European Union. All EMR and wearable sensor data are stored in pseudonymized form on secured hospital databases and are only accessible by authorized persons for this project. The questionnaire data are stored in pseudonymized form using the Research Manager platform (version 5.57.0). On signing the consent form, participants agree to the utilization of their data collected until the moment of potential withdrawal. Also, they agree to share relevant data with the involved researchers from the university or from regulatory authorities. Furthermore, patients are requested to provide consent for the utilization of their data in future studies pertaining to remote healthcare. No collection of biological specimens for storage will be conducted.

### Monitoring and dissemination

Given the previous experience with the wearable sensor [13, 14, 23], no serious adverse events or serious adverse device effects are to be expected. However, if an unanticipated serious adverse event or serious adverse device effect occurs within 30 days, the primary investigator will be notified swiftly and the incident will be documented in the patient’s EMR. In case of a safety-relevant event, the primary investigator will inform the sponsor and ethics committee of the incident within 24 h.

This clinical trial will be audited by an independent auditor to ensure the trial’s integrity. The first visit by the auditor occurs before the start of the trial. Hereafter, visits take place after the first three inclusions and every year until the trial is finished. After finishing the trial, there will be a concluding visit. During these visits, the integrity of the trial process will be assessed, and an evaluation will be conducted to ensure that the involved parties have adequately fulfilled their tasks and responsibilities. Given the perceived low-risk nature of utilizing a commercially available CE certified wearable sensor, the involvement of a data monitoring committee was deemed unnecessary. The trial steering committee, comprising representing physicians (ED, internal medicine, and anesthesiology) and researchers of different fields, will convene in monthly meetings to assess the conduct of the trial. Furthermore, daily support will be provided by two researchers, four charge nurses working in the AAW, and two ED physicians. Amendments to the protocol will be communicated with the ethical committee in accordance with the standards of the Central Committee on Research Involving Human Subjects in the Netherlands. The results will be communicated via multiple scientific publications and conference talks.

### Substudy

As a substudy of this trial, algorithms to predict whether a patient can be discharged safely from the AAW or whether a patient will deteriorate in the AAW will be developed. These predictive algorithms will consider a range of hospital data, including demographics, vital signs, laboratory results, assessments (including EWS), medication use, care activities, and logistical information. Depending on the quality and characteristics of the data, which remain unknown until the trial is conducted, various artificial intelligence techniques, such as logistic regression and Random Forest, may be employed. Hospital data from both the sensor group and control group will be used to develop the algorithms. Furthermore, for the sensor group, additional algorithms will be developed that also utilize wearable sensor data to evaluate the additional value of the sensor. Additionally, for the sensor group, predictive algorithms for patient deterioration after hospital discharge will be developed, considering wearable sensor data and patient demographics. For these reasons, the wearable sensor will not be removed after AAW discharge, but will remain attached for the full extent of time specified by the manufacturer, which is 14 days. Predictive performances of the algorithms will be assessed by the area under the receiver operator characteristic curve, sensitivity, and specificity, among others.

## Intervention

**Fig. 1:**
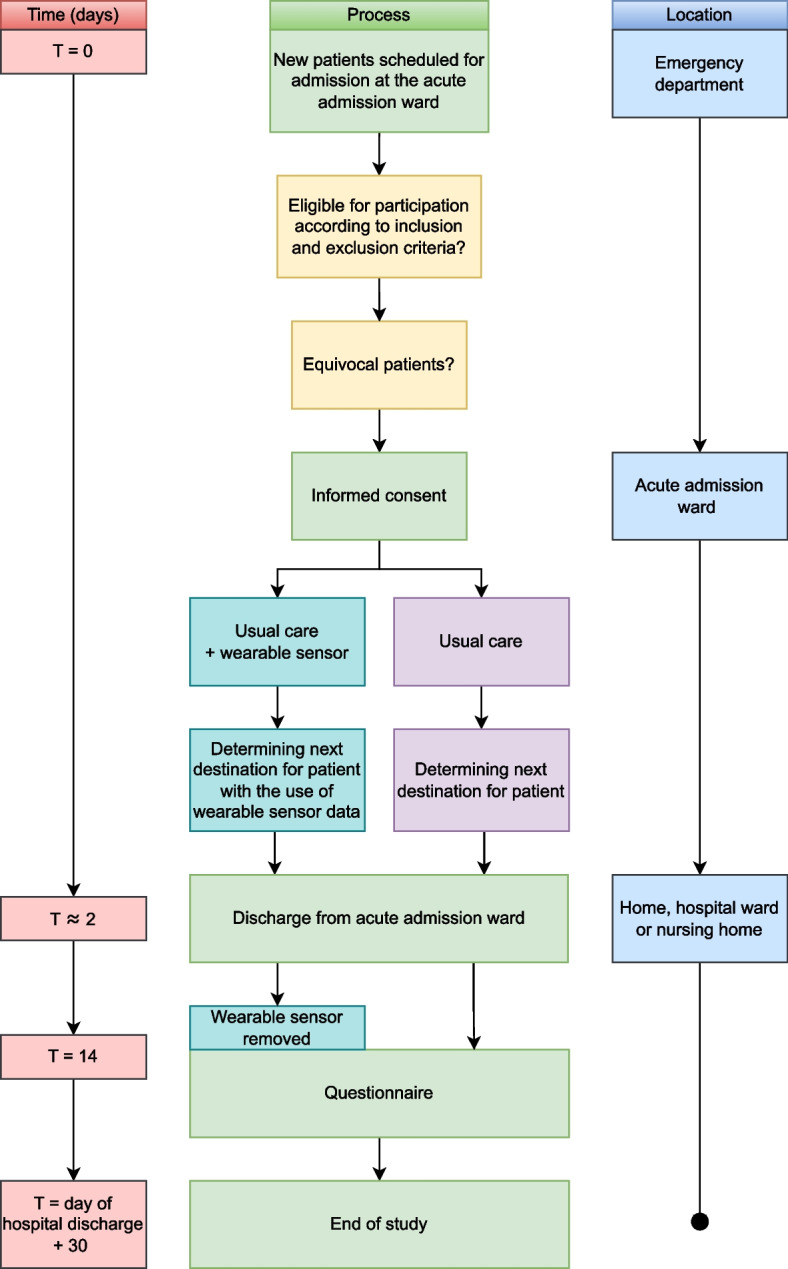
An outline of the trial pathway

To assess whether adding continuous monitoring to usual care has an effect on patient discharge, this randomized controlled trial includes a control group of patients receiving usual care alone (see Fig. 1). Usual care includes intermittent vital sign monitoring and the calculation of an EWS. This intermittent vital sign monitoring is done manually by nurses every 4 to 8 h, or more if clinically deemed necessary. Thus, patients in the control group will not receive a wearable sensor and will not be continuously monitored. For these patients, the physician only uses the information usually available to decide whether a patient can be discharged.

**Fig. 2:**
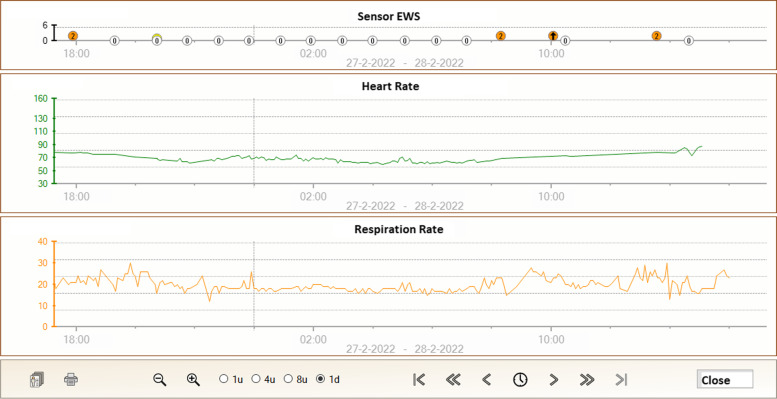
Upper graph: Sensor Early Warning Score based on heart rate (HR) and respiratory rate (RR). Center and lower graphs: HR (green) and RR (orange) of a patient, respectively

Patients in the sensor group receive a wearable sensor for continuous monitoring of HR, RR, posture, and activity by a nurse, in addition to usual care, immediately after allocation in the AAW. The data from the wearable sensor is accessible for nurses and physicians in the AAW on a computer dashboard (see Fig. 2). No alarms will be generated to avoid potential alarm fatigue. Instead, notifications of deteriorating patients are displayed on the dashboard (see the “Wearable sensor” section). During the daily bedside rounds, physicians are asked to consider the continuous monitoring data, in addition to the data usually available, in deciding whether a patient can be discharged. As there is no gold standard on how to interpret these continuous monitoring data with regard to patient discharge in such a heterogeneous population, physicians are not provided with specific instructions on how to appraise these data. However, vital signs have been demonstrated to predict patient deterioration and we therefore hypothesize that the availability of these continuous vital sign measurements will help clinical decision-making with regard to patient discharge. There are no restrictions regarding concomitant care during the trial.

After admission to the AAW, patients are either discharged home, to a nursing home, or admitted to an in-hospital ward. In all cases, for patients in the sensor group, the wearable sensor remains attached for a total of 14 days. However, after AAW stay, these measurements are no longer presented to healthcare professionals, but only analyzed retrospectively. After 14 days, the wearable sensor is removed by the patient and patients of both the control and sensor group are asked to fill in the patient questionnaire. Patient follow-up will be completed 30 days after hospital discharge.

A schedule of enrolment, interventions, and assessments of this trial is shown in SPIRIT format in Table 2 [18]. Hospital readmissions, ED revisits, and mortality are monitored until 30 days after hospital discharge. At the end of the trial, healthcare professionals are asked to fill in a questionnaire to assess satisfaction and the usability of the wearable sensor. Facilitators and barriers for implementing continuous monitoring in usual care in the AAW and at home will be assessed by patient questionnaires, healthcare professional questionnaires, and interviews with healthcare professionals.

**Table 2:**
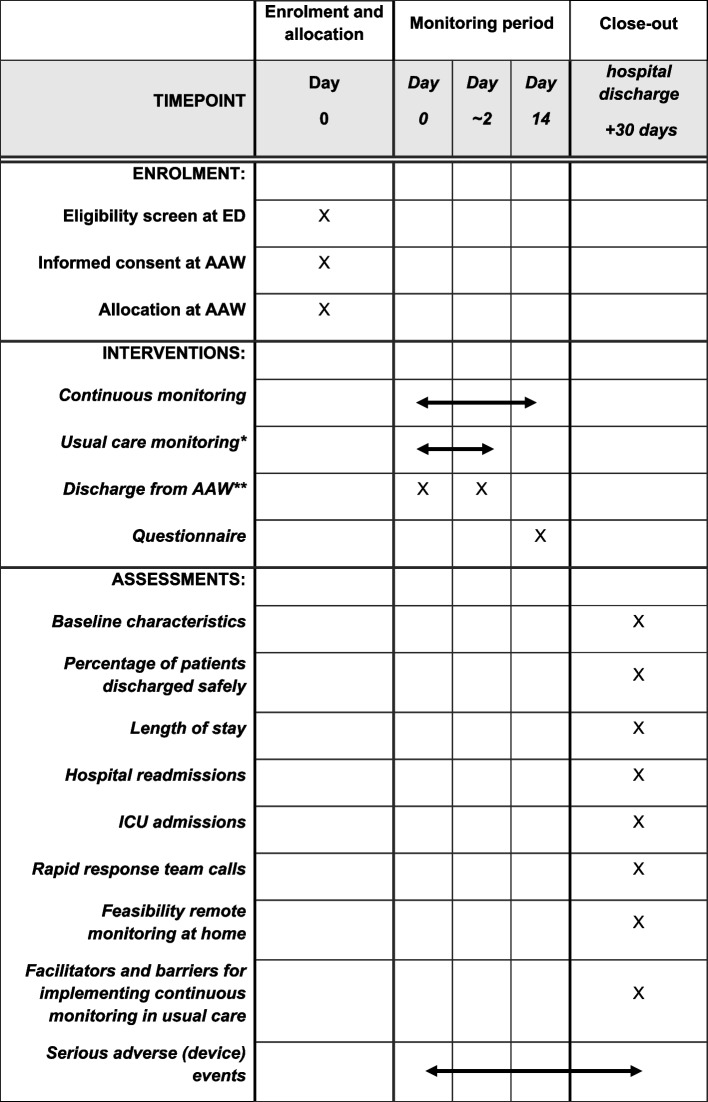
Schedule of enrolment, interventions, and assessments of this trial

^*^Takes place until acute admission ward discharge

^**^The moment of discharge is typically within 48 h, but can be later in some cases

## Discussion

This trial aims to determine the effect of continuous monitoring of vital signs on the percentage of patients that are directly discharged safely from the AAW. Continuous monitoring receives an increasing amount of attention for different groups of patients in- and outside hospitals, such as obstetric [24], bariatric surgery [23], chronic pulmonary disease [25], or heart failure patients [26], and patients admitted to medical-surgical wards [27]. For instance, it has been shown that the use of continuous monitoring of patients in medical-surgical wards reduces ICU admissions and hospital LOS [27, 28]. Furthermore, continuous monitoring decreases the risk of mortality and might decrease hospital LOS and number of rapid response team calls in non-ICU patients [29]. Such positive effects of continuous monitoring can therefore contribute to discharge optimization and increased efficacy of hospital beds. To our knowledge, the effects of introducing continuous monitoring of HR and RR in the AAW or similar wards on the discharge process have not been investigated yet.

This trial has several strengths to assess the effects of continuous monitoring. First, whereas the abovementioned studies measured the acceptability of patients and nurses [24], or have an observational [25, 26] or before-and-after study design [27, 28], this trial is designed as a Randomized Controlled Trial. Consequently, any difference in study outcome is allowed to be attributed to the intervention [30]. Another strength lies in the patient selection criteria. We aim to only include equivocal patients, as the care pathway for these patients is uncertain and the clinical usefulness of the wearable sensor is expected to have a large impact in the discharge decision. Thus, if the results are promising, standard implementation of continuous monitoring for equivocal patients in the AAW can be the next step in enhancing the discharge process.

However, this trial also has some limitations. First, insight into the continuous monitoring data to physicians and nurses is only given during AAW stay. Therefore, the additional value of the ability of the wearable sensor to monitor a patient outside the hospital will not have any influence on the decision-making in the AAW. Second, the continuous data are displayed in a separate dashboard at which physicians and nurses need to login every time they want to access the data, which might become a barrier to use the data in the discharge decision. Third, continuous data are most valuable when used for trend analysis, with which most physicians have yet limited experience. Fourth, the decision of a physician to discharge a patient is dependent on many factors other than HR and RR alone. Fifth, due to the use of a wearable sensor, blinding of patients and physicians was not feasible, except for the allocation process. This might have influenced the way patients are treated during their stay in the AAW. Thus, if different effects are found, this may not be only attributable to the sensor itself but to the overall process in the AAW, which might have changed in those patients with a wearable sensor. Last, the dashboard utilized in this study currently provides no track record about its usage. Still, all efforts (including a short survey) will be made to increase the availability and awareness of this system among clinicians.

The substudy of this trial also aims to develop predictive algorithms for deterioration and safe discharge based on artificial intelligence. Such algorithms are increasingly investigated, for example in predicting the risk of coronary syndrome [31] and heart failure [32]. For these predictions, artificial intelligence outperformed traditional methods. Moreover, artificial intelligence is also promising in combination with time series of continuous monitoring data, for example in predicting circulatory failure [33] or mortality in the ICU [34], cardiac arrest at the pediatric ICU [35], and stability at general medical wards [8]. However, to the best of our knowledge, no literature is available that uses time series data of continuous monitoring with artificial intelligence at wards such as the AAW.

In conclusion, this trial will give insights into the effects of adding continuous monitoring to usual care on the discharge process in the AAW. Also, possible facilitators and barriers of implementing this continuous monitoring will be assessed.

## Trial status

Protocol version: 2.0, 30-06-2021

Trial start date: December 6, 2021

Estimated trial completion date: June 1, 2024

## Supplementary Information

**Additional file 1.** SPIRIT checklist.

## Data Availability

The full protocol, data sets, and statistical code utilized in the present study are available from the corresponding author upon reasonable request, subject to legal and privacy considerations.

## Abbreviations

AAW: Acute admission ward
ED: Emergency Department
EMR: Electronic Medical Record
EWS: Early Warning Score
HR: Heart rate
ICU: Intensive care unit
LOS: Length of stay
RR: Respiratory rate
RRT: Rapid Response Team
